# The Anti-Vivisection Hospital

**Published:** 1909-09-25

**Authors:** 


### THE ANTI-VIVISECTION HOSPITAL.

**The following Correspondence between the Secretary of the above Institution (Mr. George W. F. Robbins) and Sir Henry Burdett, K.C.B., K.C.V.O., is reprinted from "The Times." The profession and the public by reading it will be able to understand why it is that none of the great Funds can in justice to the public accept this Institution as an ordinary hospital in present circumstances.**

I.—FROM MR. ROBBINS.

*Times*, August 28, 1909.

Sir,—The rejection of a grant of £97 10s., which was made to this hospital by the Council of the Metropolitan Hospital Sunday Fund, may seem unjustifiable to the general public. Almost universal approval has been expressed to this office by those who know all the circumstances. In particular, Battersea, the district most concerned, heartily supports the board's action. Last year (also in June this year—a fact not generally known) the distribution committee of the M.H.S.F. peremptorily informed this hospital that, unless it altered its constitution in the matter of its anti-vivisection methods, etc., it would not recommend it for a grant. To this the chairman replied that "the board of management had no intention of recommending any alteration in our constitution in the essential and particular design of the institution as a general hospital for medical and surgical treatment from which 'vivisection' (as defined by the Royal Commission of 1876) is utterly banished." A reply was received as follows: "The terms of your letter make it impossible for the committee to recommend an award being made to your hospital."

At the recent Mansion House meeting, Sir Henry Burdett, K.C.B. (after accusing this hospital of "pretentious humbug," and making a damaging assertion, for the proof of which I am still awaiting his convenience), supported the vote with these significant words, "In giving the grant it should go forth that . . . it was given in the hope that the authorities would mend their ways, purge their methods, and, in fact, fall into line with all the best administered hospitals in the country."

The hospital board, therefore, would, I submit, by any acceptance of a grant so awarded, have not easily escaped from a serious charge of entertaining, *ipso facto*, a possibility of such a "falling into line" (undoubtedly "Burdettese" for tearing up its anti-vivisection constitution), or of accepting the grant under false pretences.

To the Court of Governors of the hospital—which is, in fact, as a body, totally opposed to vivisection in every form—it has to account for its action and for any suspicion of faltering in its principles.

Add to this the other damaging and undoubtedly insulting remarks of Sir William Church, Mr. Thomas Bryant, F.R.C.S., Sir Henry Burdett, and the Rev. J. Grundy, and the reply to the M.H.S.F. assumes another and an intelligible aspect. It becomes, not merely justifiable, but necessarily straightforward and dignified. The board fully realised the responsibility of refusing any money given for relief of suffering. It did not believe that the poor of Battersea would eventually suffer. In this view the moral and financial support which has already come to hand has fully confirmed it.

Yours,
George W. F. Robbins, Secretary.
National Anti-Vivisection Hospital and Battersea General Hospital, Battersea Park, S.W.

II.—FROM SIR H. BURDETT.

*Times*, September 10, 1909.

Sir,—Being engaged at the moment on a tour of inspection of the principal hospitals throughout the country, I have only to-day had an opportunity to consider the letter from Mr. Robbins which you published on the 28th ult. My statement at the Mansion House was a matter of fact, not opinion. It was that the medical profession knew perfectly well that in the treatment of disease the Anti-Vivisection Hospital must employ remedies which would not have been available had it not been for the discoveries resulting from vivisection. It is pretentious humbug for any institution to claim to be an up-to-date modern hospital, worthy of public confidence, if the management exclude the application of all such remedies in the treatment of disease. What those remedies are the evidence given before the Commission now sitting, which has been published, clearly demonstrates.

Mr. Robbins invited me to name these remedies, it is true; but that I am not called upon to do, for the evidence referred to is authoritative and convincing. Anyway, the fact of the knowledge possessed by the medical profession, to which I referred in my speech, places no obligation on myself to name remedies and so enable Mr. Robbins to attempt to draw a herring across the scent. Either the Anti-Vivisection Hospital does employ every remedy in the treatment of disease prescribed in a modern up-to-date hospital, or it does not. If it does, then it is pretentious humbug for its managers to call it an anti-vivisection hospital conducted on purely anti-vivisectionist principles. If it does not, and the aseptic system is not strictly carried out in its surgical wards, it is pretentious humbug for the authorities to call it a hospital at all.

Further, as I have stated in my correspondence with Mr. Robbins, published in The Hospital of the 28th ult., until an authoritative statement is forthcoming as to the adoption or non-adoption of the aseptic treatment at this hospital, no prudent person of either sex can surely attend at the Anti-Vivisection Hospital as a patient, or permit any member of their family to do so. The Hospital points out: "That this institution does not admit diphtheria cases, and would not give them antitoxin, may be gathered by the evidence given by one of the staff before the Royal Commission." Indeed, the senior physician of this institution about a year and a half ago called diphtheria antitoxin "animal filth" at a large public meeting in the Battersea Town Hall, as reported in the *Times*.

Any who may wish for some examples of pretentious humbug should obtain a copy of the report of the Anti-Vivisection Hospital for 1909 and study it carefully.

I am, Sir, yours faithfully,

Cardiff, September 5. Henry C. Burdett.

III.—FROM MR. ROBBINS.

*Times*, September 16, 1909.

Sir,—Sir Henry Burdett's letter in your issue of September 10 is not to the point at issue between us. He has simply to answer my challenge. Until he does so he must not be surprised if we consider him a negligible quantity in matters referring to this hospital. It is Sir Henry, not I, who is drawing the red herring across the scent.

Here is the clear issue: Sir Henry stated that this "hospital must employ remedies which would not have been available had it not been for the discoveries resulting from vivisection."

He has declared on paper that this is "demonstrably true" and that he "can prove it without my help." Mere assertion will not help him with me. Moreover, he cannot remove his personal responsibility to the Royal Commission, the report of which is not yet to hand, and which does not contain one single piece of published evidence which can help Sir Henry in his dilemma. If Sir Henry replied at all to my challenge, he was bound to name a remedy "which, etc., etc.," for that is my challenge.

My last letter was solely to explain to your readers why the Metropolitan Hospital Sunday Fund award was rejected, and I do not propose to trespass further on your courtesy.

Yours faithfully,
George W. F. Robbins, Secretary.
National Anti-Vivisection Hospital and Battersea General Hospital, Battersea Park, S.W., September 13.

IV.—FROM SIR H. BURDETT.

*Times*, September 17, 1909.

Sir,—Mr. Robbins's letter in the *Times* of to-day demonstrates the difference between the policy of the Anti-Vivisection Hospital and all other hospitals. Every other hospital makes the claims of the patients the first consideration in everything. Mr. Robbins ignores the whole of the issues raised in my letter of the 5th inst., which vitally affect every patient attending this hospital. Mr. Robbins misquotes my speech directly and my letter indirectly. Using this direct misquotation which converts a fact into an expression of opinion, he waves the word "challenge" above his head and runs away.

This will not do at all. The evidence before the Royal Commission has been published, and proves my statement to the hilt. That statement was that the medical profession knew perfectly well that in the treatment of disease the Anti-Vivisection Hospital must employ remedies which would not have been available had it not been for the discoveries resulting from vivisection.

Every other hospital in the country employs these remedies.

If the above hospital employs them, it is pretentious humbug to call it "The Anti-Vivisection Hospital." If it does not, it is not a hospital and ought not to be regarded as a hospital by the great funds. Indeed, if the staff of the Anti-Vivisection Hospital avoid the use of all methods of treatment which are employed as the result of experiments, they are guilty of infinitely more cruelty to their patients than any vivisector would dare to inflict upon an animal.

I am, Sir, your obedient servant,

Henry C. Burdett.

Wolverhampton, September 16.

